# Linear brain measurement: a new screening method for cognitive impairment in elderly patients with cerebral small vessel disease

**DOI:** 10.3389/fneur.2024.1297076

**Published:** 2024-01-22

**Authors:** Jing Wang, Jinhua Lu, Mingqing He, Ziyang Song, Lingyan Dong, Haiying Tang, Yueju Wang, Zheping Zhou

**Affiliations:** ^1^Department of Geratology, The First Affiliated Hospital of Soochow University, Suzhou, China; ^2^Department of Radiology, Dushu Lake Hospital Affiliated to Soochow University, Suzhou, China; ^3^Department of Geratology, Affiliated Changshu Hospital of Nantong University, Changshu, China

**Keywords:** cerebral small vessel disease, elderly patients, cognitive impairment, linear measurement, imaging

## Abstract

**Background:**

The old adults have high incidence of cognitive impairment, especially in patients with cerebral small vessel disease (CSVD). Cognitive impairment is not easy to be detected in such populations. We aimed to develop clinical prediction models for different degrees of cognitive impairments in elderly CSVD patients based on conventional imaging and clinical data to determine the better indicators for assessing cognitive function in the CSVD elderly.

**Methods:**

210 CSVD patients were screened out by the evaluation of Magnetic Resonance Imaging (MRI). Then, participants were divided into the following three groups according to the cognitive assessment results: control, mild cognitive impairment (MCI), and dementia groups. Clinical data were collected from all patients, including demographic data, biochemical indicators, carotid ultrasound, transcranial Doppler (TCD) indicators, and linear measurement parameters based on MRI.

**Results:**

Our results showed that the brain atrophy and vascular lesions developed progressive worsening with increased degree of cognitive impairment. Crouse score and Interuncal distance/Bitemporal distance (IUD/BTD) were independent risk factors for MCI in CSVD patients, and independent risk factors for dementia in CSVD were Crouse Score, the pulsatility index of the middle cerebral artery (MCAPI), IUD/BTD, and Sylvian fissure ratio (SFR). Overall, the parameters with high performance were the IUD/BTD (OR 2.28; 95% CI 1.26–4.10) and SFR (OR 3.28; 95% CI 1.54–6.91), and the AUC (area under the curve) in distinguishing between CSVD older adults with MCI and with dementia was 0.675 and 0.724, respectively. Linear brain measurement parameters had larger observed effect than other indexes to identify cognitive impairments in CSVD patients.

**Conclusion:**

This study shows that IUD/BTD and SFR are good predictors of cognitive impairments in CSVD elderly. Linear brain measurement showed a good predictive power for identifying MCI and dementia in elderly subjects with CSVD. Linear brain measurement could be a more suitable and novel method for screening cognitive impairment in aged CSVD patients in primary healthcare facilities, and worth further promotion among the rural population.

## 1 Introduction

Cerebral small vessel disease (CSVD) is the most common cause of vascular cognitive impairment and dementia in the elderly population, this disease imposes a substantial burden on families and societies (1). The reduction of cognitive function in CSVD patients are correlated with lacunar infarcts, white matter lesions, microbleeds and always contributes to a high risk for adverse clinical outcomes such as falls, fractures, hospitalization and even death (2). In addition, the older adults constitute the most extensive and fastest-growing population with dementia (3). Therefore, old CSVD patients with cognitive impairment need much weighted attention.

However, the performances of cognitive declines are always inconsistent in elderly CSVD patients and it is difficult to find population with cognitive impairments in CSVD patients due to few subjective symptoms (4). Meanwhile, imaging technique often be used for diagnosis of CSVD but rarely for cognitive impairments (5). The poor compliance and little attention for screening of dementia among the older patients (or their families) may lead to a low detection rate of cognitive impairment. Traditional cognitive screening assessments by face-to-face conversation with elderly patients are laborious and time-consuming with limited sensitivity (6). Therefore, there is a critical need for timesaving and accurate screening methods to detect and diagnose cognitive impairments in elderly CSVD patients.

Greater multimodal and advanced magnetic resonance imaging (MRI) technologies including brain volume, diffusion tensor imaging (DTI), functional magnetic resonance imaging (fMRI) have been applied in the evaluation of cognitive function of CSVD patients (7). Screening test for cognitive impairment in elderly CSVD patients by means of imaging techniques is a promising approach. There are several recognized structural markers of CSVD on MRI, including white matter hyperintensities (WMH), lacunes, and brain atrophy (8). These individual imaging features of CSVD are inter-related, and their respective correlations with cognition have been demonstrated (9). DTI parameters and brain network measures have also been surrogate marker as cognitive impairment (10). However, the consistency between these imaging markers and the degree of cognitive impairment remains to be established. Additionally, Advanced imaging equipment is technically demanding and relatively expensive, so cannot be used widely in the community and township hospitals in China.

Previous brain imaging studies have revealed the linear changes of brain volume in Alzheimer disease (11), but studies on this aspect in CSVD patients are still rare. The latest research has showed that Sylvian fissure ratio is valuable for cognitive decline screening (12). This linear measurement method is intuitive and easy to learn without requiring additional software and devices. In such situations, linear measurements based on CT or MRI can deliver highly useful information, which are cost-saving and convenient handling. This novel approach is expected to be promoted in community settings and care homes in the future.

Therefore, the present study’s main aims were to construct clinical prediction models for different degrees of cognitive impairments in elderly CSVD patients to determine the more objective, convenient and sensitive indicators for assessing cognitive function. In addition, by analyzing common examination data related to risk factors, we expect to derive valuable screening index that can predict the occurrence of different degrees of cognitive impairment in CSVD simply and effectively.

## 2 Methods

### 2.1 Study design and participants

300 older patients aged 75–95 years were invited to participate in the cross-sectional study organized by the memory disorder clinic of the geriatric department in First Affiliated Hospital of the Soochow University, and Ethical approval was obtained from the Ethical committee of the First Affiliated Hospital of Soochow University. All patients and their caregivers provided written informed consent before participating in the study. The inclusion criteria were as follows: (1) Age was older than 65 years. (2) With complaints of subjective cognitive decline. The following patients were excluded: (1) Patients with other disorders that may affect cognitive function such as poisoning, infection, Parkinson disease, immune demyelination. (2) Patients with intracranial hemorrhage, intracranial space-occupying lesion, large cerebral infarction, and hydrocephalus. (3) Patients with visual or hearing impairment or language barrier which may affect their cooperation in the test. (4) Patients with severe conditions such as cardiac failure, renal and liver dysfunction. All participants received a comprehensive clinical evaluation that included a detailed demographic and medical history, appropriate laboratory tests, a battery of neuropsychological tests, carotid ultrasound, TCD and cranial MRI scan.

Finally, 210 participants were included in the study, which were diagnosed with CSVD according to the following criteria.

### 2.2 Diagnosis of CSVD

We adopted the Standards for Reporting Vascular changes on nEuroimaging (STRIVE) criteria for the diagnosis of the CSVD (13). The diagnosis of CSVD contains at least one of the following neuroimaging markers: (1) Lacunar infarction, which includes recent small subcortical infarct (RSSI) and lacune of presumed vascular origin. (2) White matter hyperintensity of presumed vascular origin, which defined as punctate, patchy, or confluent hyperintense signal on T2-weighted and FLAIR sequences, and isointensity or hypointensity signal on T1-weighted sequence. The hyperintense signal in subcortical gray matter and brainstem is not included. (3) Cerebral microbleed, which defined as small hypointensity signal with diameter ranging from 2 to 10 mm on susceptibility weighted imaging but no corresponding signal on FLAIR, T1-weighted, or T2-weighted sequences. (4) Perivascular space, which defined as round (axial) or linear (parallel to vessels) fluid-filled space with diameter of less than 3 mm.

### 2.3 Medical and cognitive assessments

The Beijing edition of the Motreal Cognitive Assessment (MoCA-BJ) was used to evaluate cognitive function of participants (14). The Clinical Dementia Rating scale (CDR) was also performed at all participants to characterize 6 domains of cognitive and functional performance including memory, orientation, judgment and problem solving, community affairs, home and hobbies, and personal care. Each domain is rated on 5-point scale independently from each other (0, 0.5, 1, 2, or 3) (15), The CDR-SB is calculated by adding the score from each domain box score and the score ranges from 0 (no impairment) to 18 (severe impairment) (16). Trained neuropsychologists assessed all the scales. The clinical and demographic assessment included: age, sex, education, presence of hypertension (HP), diabetes mellitus (DM), and coronary heart disease (CHD). Recent laboratory findings were collected by researchers with qualitative experience, including Total cholesterol (TC), triglyceride (TG), high-density lipoprotein cholesterol (HDL), low-density lipoprotein cholesterol (LDL), apolipoprotein A (APO-A), apolipoprotein B (APO-B), apolipoprotein A1/B (APO-A1/B), Lipoprotein(a) [Lp(a)], creatinine (Cr), uric acid (UA), and high-sensitivity C-reactive protein (hsCRP).

### 2.4 Linear measurement based on MRI

**Figure 1:**
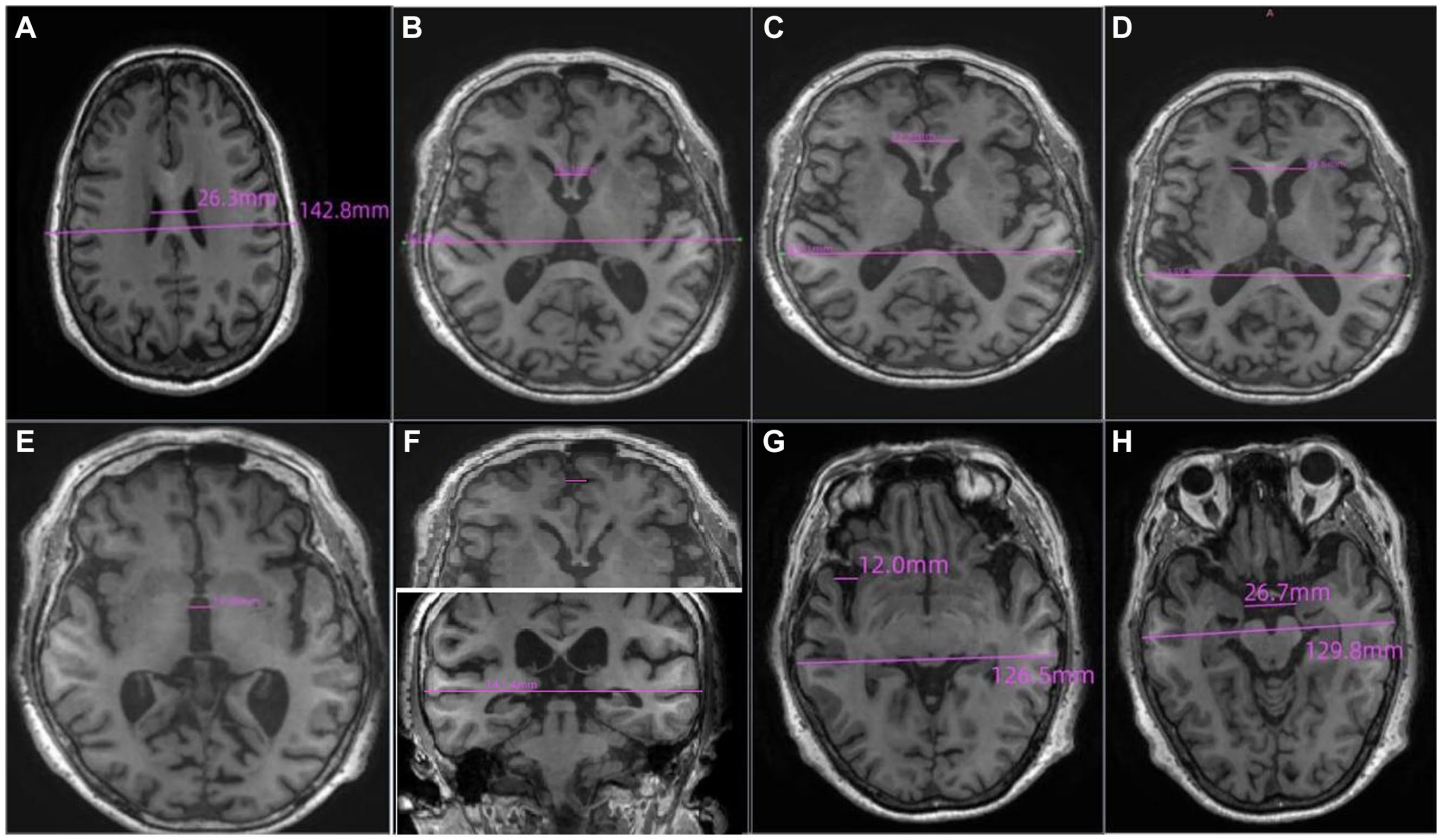
The definition of the linear measurements of MRI scans. **(A)** Lateral ventricular body width index (VBWI), **(B)** Bicaudate ratio (BCR), **(C)** Bifrontal ratio (BFR), **(D)** The Evans index (EI), **(E)** The maximum width of the third ventricle (MTV), **(F)** Frontal interhemispheric fissure ratio (FFR), **(G)**. Sylvian fissure ratio (SFR), **(H)** Interuncal distance/Bitemporal distance (IUD/BTD).

The brain’s imaging was performed with a 3.0 T MRI scanner which included axial T1-weighted, axial T2-weighted, and fluid-attenuated inversion recovery (FLAIR). All image acquisition and analysis were performed by two radiologists. As shown in Figure 1, the linear measurements of MRI scans were defined as follows:

- Lateral ventricular body width index (VBWI): The internal transverse diameter of the skull perceived as the maximum diameter by the radiologist divided by the greatest distance between the outside lateral walls of lateral ventricle body.
- Bicaudate ratio (BCR): The axial slice on which the caudate nuclei produced the greatest amount of indentation on the lateral ventricles was identified, and the distance between the two caudate apices was measured. This value was divided by the maximum width of the skull at the same level as the caudate measurement.
- Bifrontal ratio (BFR): Measured on the slice best showing the caudate nuclei. It is the distance between the tips of the frontal horns divided by the distance between the skull’s inner tables along the same line.
- The Evans index (EI): The ratio of the transverse diameter of the anterior horns of the lateral ventricles to the greatest internal diameter of the skull.
- The maximum width of the third ventricle (MTV): The radiologist identified the five largest brain cross sections of the third ventricle and then utilize an image computer program to measure the width of the third ventricle. The maximum value obtained from these measurements should be recorded.
- Frontal interhemispheric fissure ratio (FFR): The maximal width of the interhemispheric fissure from the slices demonstrating the frontal lobes judged by the radiologist divided by the trans-pineal coronal inner table diameter.
- Sylvian fissure ratio (SFR): The average of the maximal Sylvian widths taken from the cut showing the widest Sylvian fissure divided by the transpineal coronal inner table diameter.
- Interuncal distance/Bitemporal distance (IUD/BTD): The ratio of the distance between the unci of temporal lobes/the distance between outer margins of temporal lobes at the level where IUDs were obtained.

### 2.5 Acquisition of carotid ultrasound and TCD index

The carotid ultrasound measurements were performed on a Philips CX50 ultrasound instrument. Two sonographers record ultrasound images. Crouse score was used to assess the severity of carotid and coronary atherosclerosis (17). It was defined as the sum of maximum thicknesses of all plaques. A Multi-Dop FDT98 type TCD analyzer was used for TCD detection. The supine position was used during the detection. The probe was placed in the preauricular and occipital region of the temporal window to detect the blood flow velocity and the PI (pulsatility index) in the distal segment of the extracranial internal carotid arteries (ICA), middle cerebral artery (MCA), anterior cerebral artery (ACA), posterior cerebral artery (PCA), vertebral artery (VA) and basilar artery (BA). The parameters were obtained as the mean of two measurements on each side.

### 2.6 Patients grouping

In our study group, according to the MoCA and CDR-SB scores (18, 19), we divided these 210 CSVD patients into three groups: control group (56 patients), CSVD without cognitive impairment: MoCA scores >24 (CDR-SB = 0); CSVD with mild cognitive impairment (MCI) group (60 patients): the MoCA scores ranges from 18 to 24 (CDR-SB, 0.5–4); CSVD with dementia group (94 patients): the MoCA scores ≤17(CDR-SB ≥ 4.5).

### 2.7 Statistical analysis

The statistical analyses were performed in SPSS version 23.0 (IBM Corp, Armonk, NY, USA), and graphics produced with R software (version 3.4.3). For all analyses, statistical significance was set at *p* < 0.05. The Skewness/Kurtosis test was used to test for normal distributions of continuous variables. Continuous variables are presented as mean ± standard deviation. Categorical variables are presented as proportions. Differences between the groups were assessed using the chi-square test, *t*-test, or Wilcoxon rank-sum test. The assumption of linearity in the logit for the continuous variable was assessed. The significance of each variable in the group was assessed by univariate logistic regression to determine the independent risk factors for different degrees of cognitive impairment in CSVD. All variables with *p* < 0.05 in the univariate logistic analyses were further assessed by multivariable logistic regression using backward stepwise selection. Two clinical prediction nomograms and forest maps to assess the risk of cognitive impairment in CSVD patients were constructed based on the results from the final multivariable logistic regression using the R software. Multicollinearity was checked before determining the final model. In addition, the nomogram was subjected to 1,000 bootstrap resamples for internal validation to assess predictive accuracy. The receiver operating characteristics (ROCs) curve was conducted using the R software to determine the nomogram’s clinical utility and single indicator.

## 3 Results

### 3.1 Participant characteristics

Table 1 shows the demographic characteristics and clinical data of 210 participants. According to the MoCA-BJ and CDR-SB criteria, 56 participants were assigned to the control group, 60 were divided to the MCI group, while 94 cases were classified as dementia. The control group, MCI group and the dementia group did not differ significantly with respect to age, sex, education level, history of DM and CHD (*p* > 0.05). Fewer patients without cognitive impairment had a history of hypertension (75.0%) compared to (92.6%) those diagnosed with dementia (*p* < 0.05). Undoubtedly, CSVD patients without cognitive impairment had higher MoCA scores (26.71 ± 1.70 vs. 19.67 ± 4.55 vs. 12.67 ± 5.67, *p* < 0.05) and lower CDR-SB scores (0.00 ± 0.00 vs. 1.51 ± 0.73 vs. 8.12 ± 3.08, *p* < 0.05). Concerning laboratory data, the dementia group had lower levels of Low-density lipoprotein cholesterol (2.31 ± 0.66 vs. 2.54 ± 0.64, *p* < 0.05) compared with the control group.

**Table 1:** Clinical characteristics and laboratory data of study participants.

Variables	Control(*n* = 56)	MCI(*n* = 60)	dementia(*n* = 94)	*p* value	
				*p*^a^	*p*^b^
Clinical and demographic data					
Age (years)	81.89 ± 3.72	82.10 ± 4.88	83.12 ± 5.09	0.813	0.124
Male (%)	71.4	70.0	77.7	0.866	0.392
Education level (years)	9.62 ± 2.56	8.9 ± 2.34	9.45 ± 2.06	0.322	0.964
MoCA	26.71 ± 1.70	19.67 ± 1.55	12.67 ± 4.27	<0.001*	<0.001*
CDR-SB	0.00 ± 0.00	1.51 ± 0.73	8.12 ± 3.08	<0.001*	<0.001*
HP (%)	75.0	80.0	92.6	0.519	0.003*
DM (%)	32.1	43.3	46.8	0.215	0.078
CHD (%)	36.6	25.0	24.5	0.826	0.752
Laboratory data					
TC (mmol/L)	4.25 ± 0.76	4.33 ± 0.94	4.03 ± 0.85	0.594	0.148
TG (mmol/L)	1.41 ± 0.81	1.34 ± 0.71	1.39 ± 0.79	0.598	0.883
HDL (mmol/L)	1.21 ± 0.28	1.23 ± 0.30	1.15 ± 0.35	0.734	0.296
LDL (mmol/L)	2.54 ± 0.64	2.59 ± 0.77	2.31 ± 0.66	0.697	0.049*
APO-A (g/L)	1.42 ± 0.24	1.36 ± 0.21	1.39 ± 0.90	0.628	0.829
APO-B (g/L)	0.90 ± 0.23	0.89 ± 0.20	0.86 ± 0.21	0.722	0.169
Lp(a) (mg/L)	146.77 ± 151.85	186.30 ± 172.51	150.87 ± 168.13	0.199	0.883
APO-A1/B	2.06 ± 1.96	2.01 ± 2.17	2.82 ± 3.77	0.999	0.285
Cr (umol/L)	79.97 ± 20.61	83.25 ± 33.48	91.14 ± 41.98	0.892	0.091
UA (umol/L)	348.47 ± 101.27	349.81 ± 96.36	347.57 ± 119.92	0.947	0.961
hsCRP (mg/L)	2.72 ± 3.31	3.91 ± 5.72	4.87 ± 13.68	0.515	0.195

Data are expressed as mean standard deviations (SD) or patient numbers (%). MCI = mild cognitive impairment. *p*^a^, MCI group compared with control group. *p*^b^, dementia group compared with control group. *p < 0.05.

### 3.2 Imaging data of study participants

As shown in Table 2 patients with MCI had a higher level of the Crouse score (5.50 ± 6.13 vs. 3.09 ± 3.65, *p* < 0.05) and IUD/BTD (0.210 ± 0.023 vs. 0.185 ± 0.023, *p* < 0.01) than the control group.

**Table 2:** Imaging data of study participants.

Variables	Control(*n* = 56)	MCI(*n* = 60)	dementia(*n* = 94)	*p* value	
				*p*^a^	*p*^b^
Carotid ultrasound and TCD data					
Crouse score	3.09 ± 3.65	5.50 ± 6.13	7.54 ± 6.62	0.033*	<0.001*
ICAPI	1.13 ± 0.47	1.12 ± 0.55	1.09 ± 0.55	0.932	0.657
MCAPI	1.09 ± 0.12	1.11 ± 0.14	1.15 ± 0.19	0.519	0.033*
ACAPI	1.12 ± 0.23	1.14 ± 0.17	1.12 ± 0.27	0.579	0.860
PCAPI	1.11 ± 0.20	1.16 ± 0.17	1.14 ± 0.24	0.252	0.507
VAPI	1.23 ± 0.26	1.31 ± 0.39	1.23 ± 0.23	0.197	1.000
BAPI	1.22 ± 0.21	1.28 ± 0.23	1.28 ± 0.23	0.218	0.133
Linear measurements of MRI scans					
VBWI	4.167 ± 0.867	3.967 ± 0.850	3.697 ± 0.702	0.174	0.001*
BCR	0.179 ± 0.053	0.188 ± 0.733	0.194 ± 0.054	0.433	0.162
BFR	0.304 ± 0.504	0.308 ± 0.047	0.320 ± 0.474	0.644	0.049*
EI	0.253 ± 0.034	0.249 ± 0.046	0.262 ± 0.032	0.621	0.156
MTV (mm)	7.947 ± 2.663	8.799 ± 2.677	9.945 ± 2.978	0.105	<0.001*
FFR	0.038 ± 0.031	0.467 ± 0.039	0.048 ± 0.032	0.159	0.078
SFR	0.061 ± 0.043	0.068 ± 0.495	0.096 ± 0.498	0.422	<0.001 *
IUD/BTD	0.185 ± 0.023	0.210 ± 0.023	0.209 ± 0.032	<0.001 *	<0.001*

Data are expressed as mean standard deviations (SD) or patient numbers (%). ICAPI = the pulsatility index (PI) of the anterior cerebral artery; MCAPI = the PI of the middle cerebral artery; ACAPI = the PI of the anterior cerebral artery; PCAPI = the PI of the posterior cerebral artery; VAPI = the PI of the vertebral artery; BAPI = the PI of the basilar artery; *p*^a^, MCI group compared with control group. *p*^b^, dementia group compared with control group. *p < 0.05.

The participants with dementia had higher levels of the Crouse score (7.54 ± 6.62 vs. 3.09 ± 3.65, *p* < 0.05), MCAPI (1.15 ± 0.19 vs. 1.09 ± 0.12, *p* < 0.05), BFR (0.320 ± 0.474 vs. 0.304 ± 0.504, *p* < 0.05), MTV (9.945 ± 2.978 vs. 7.947 ± 2.663, *p* < 0.05), SFR (0.096 ± 0.498 vs. 0.061 ± 0.043, *p* < 0.05) and IUD/BTD (0.209 ± 0.032 vs. 0.185 ± 0.023, *p* < 0.05) compared to participants without cognitive impairment. Moreover, the VBWI of CSVD patients with dementia were significantly lower than the control group (3.697 ± 0.702 vs. 4.167 ± 0.867, *p* < 0.05).

### 3.3 Multiple logistics regression

**Figure 2:**
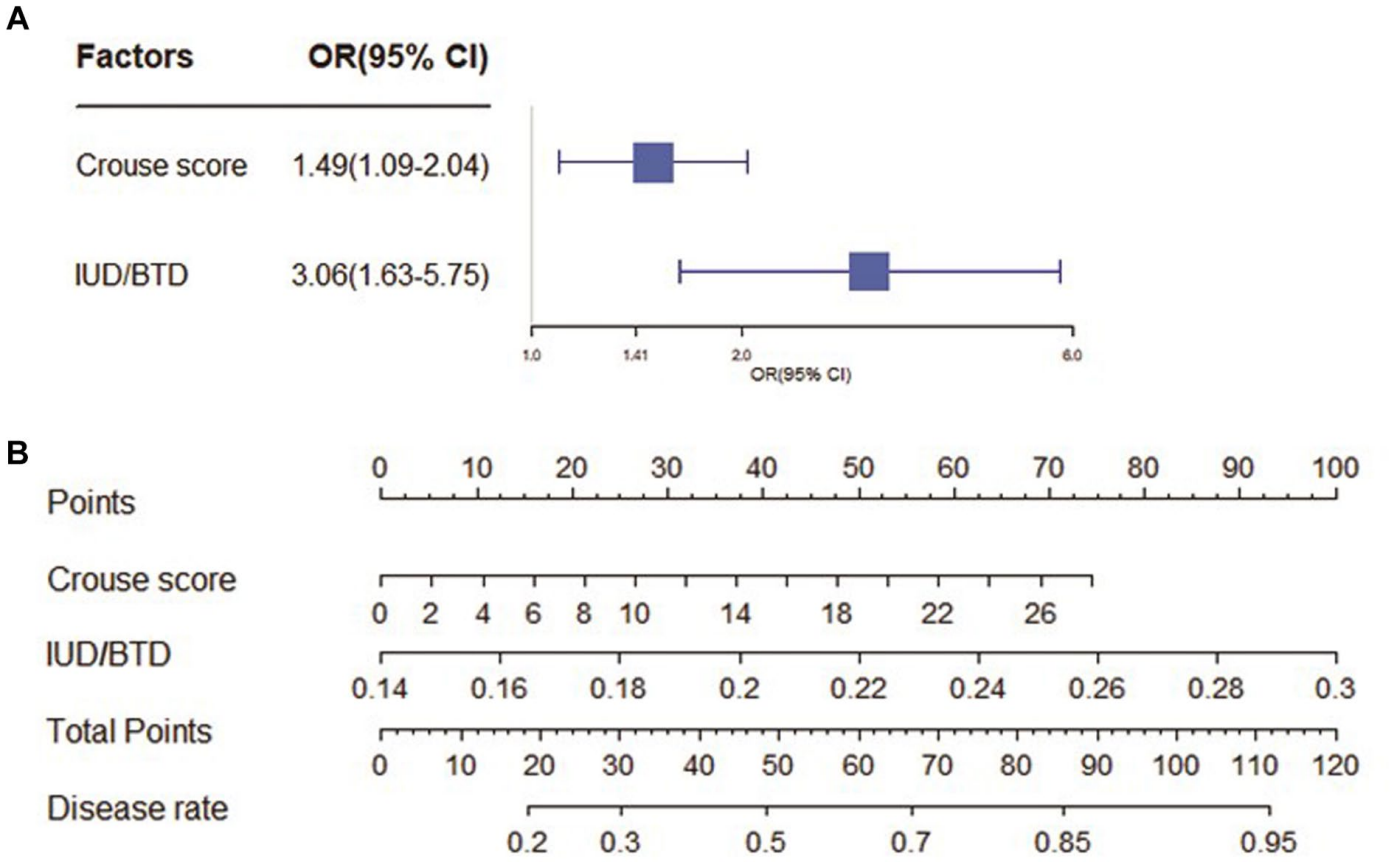
The forest map and nomogram for CSVD with MCI. **(A)** The forest plot for prediction of the occurrence of mild cognitive impairment in CSVD older adults and its predictive performance. The forest map indicated independent risk factors for CSVD with mild cognitive impairment. **(B)** The nomogram to predict probability of mild cognitive impairment with two available factors, including crouse score and IUD/BTD.

**Figure 3:**
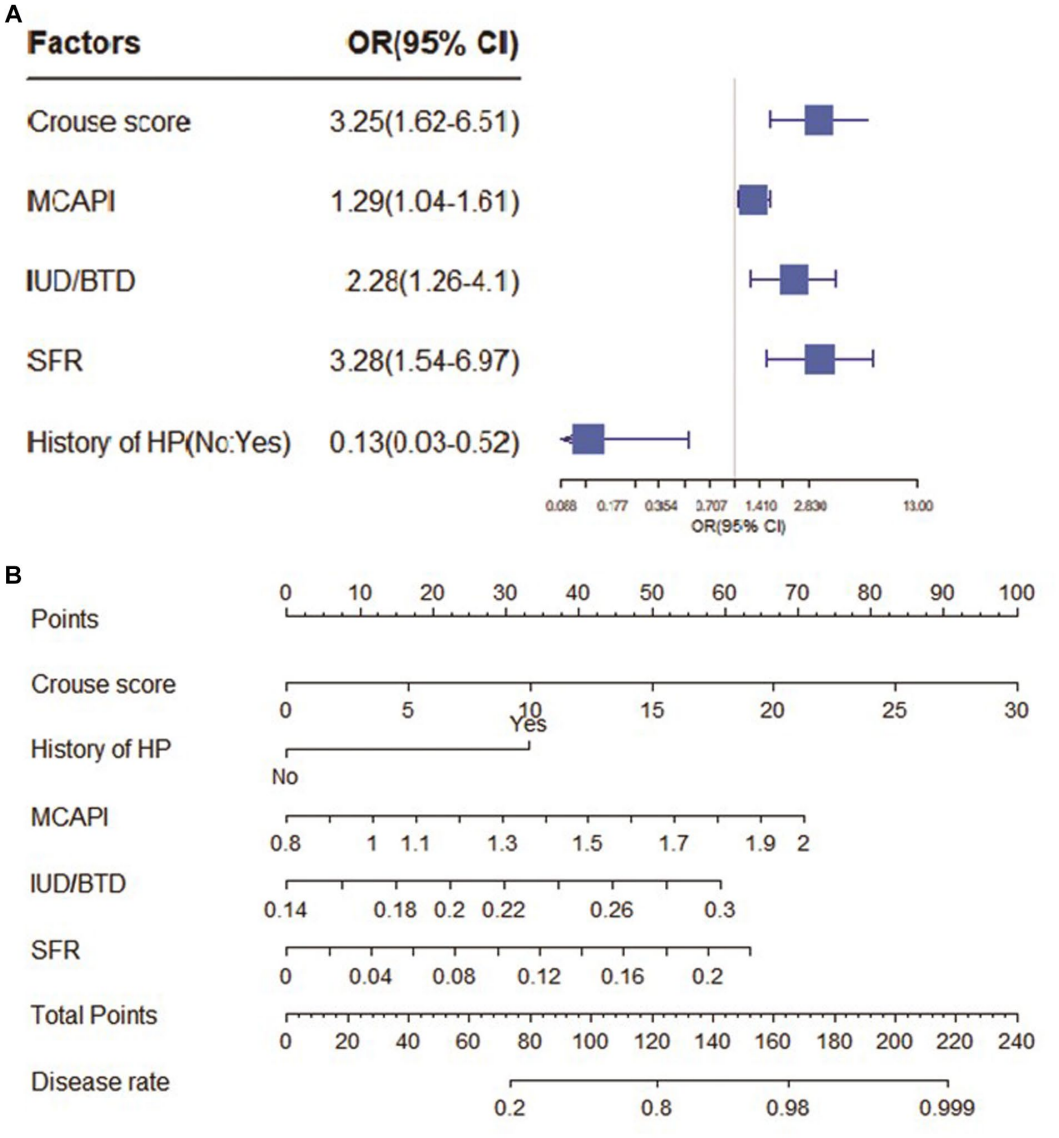
The forest map and nomogram for CSVD with dementia. **(A)** The forest plot for prediction of the occurrence of dementia in CSVD older adults and its predictive performance. The forest map indicated independent risk factors for CSVD with dementia. **(B)** The nomogram to predict probability of dementia with five available factors, including crouse score, HP, MCAPI, IUD/BTD and SFR.

As shown in Figure 2. In the CSVD with MCI group, Crouse scores (OR 1.49; 95% CI 1.09–2.04) and IUD/BTD (OR 3.06; 95% CI 1.63–5.75) were suited for the logistic regression model, the forest map and nomogram were constructed based on these two independent factors. Meanwhile, Crouse score (OR 3.25; 95% CI 1.62–6.51), history of HP (OR 0.13; 95% CI 0.03–0.52), MCAPI (OR 1.29; 95% CI 1.04–1.61), IUD/BTD (OR 2.28; 95% CI 1.26–4.10), and SFR (OR 3.28; 95% CI 1.54–6.91) were identified as factors significantly associated with CSVD with dementia in multivariate analysis, the forest map and nomogram are presented in Figure 3. Each level of every variable was assigned a score on the points scale. By adding the scores for all of the selected variables, the total score was obtained. The prediction corresponding to this total score then helped in estimating the occurrence rate for each patient.

### 3.4 ROC curve

**Figure 4:**
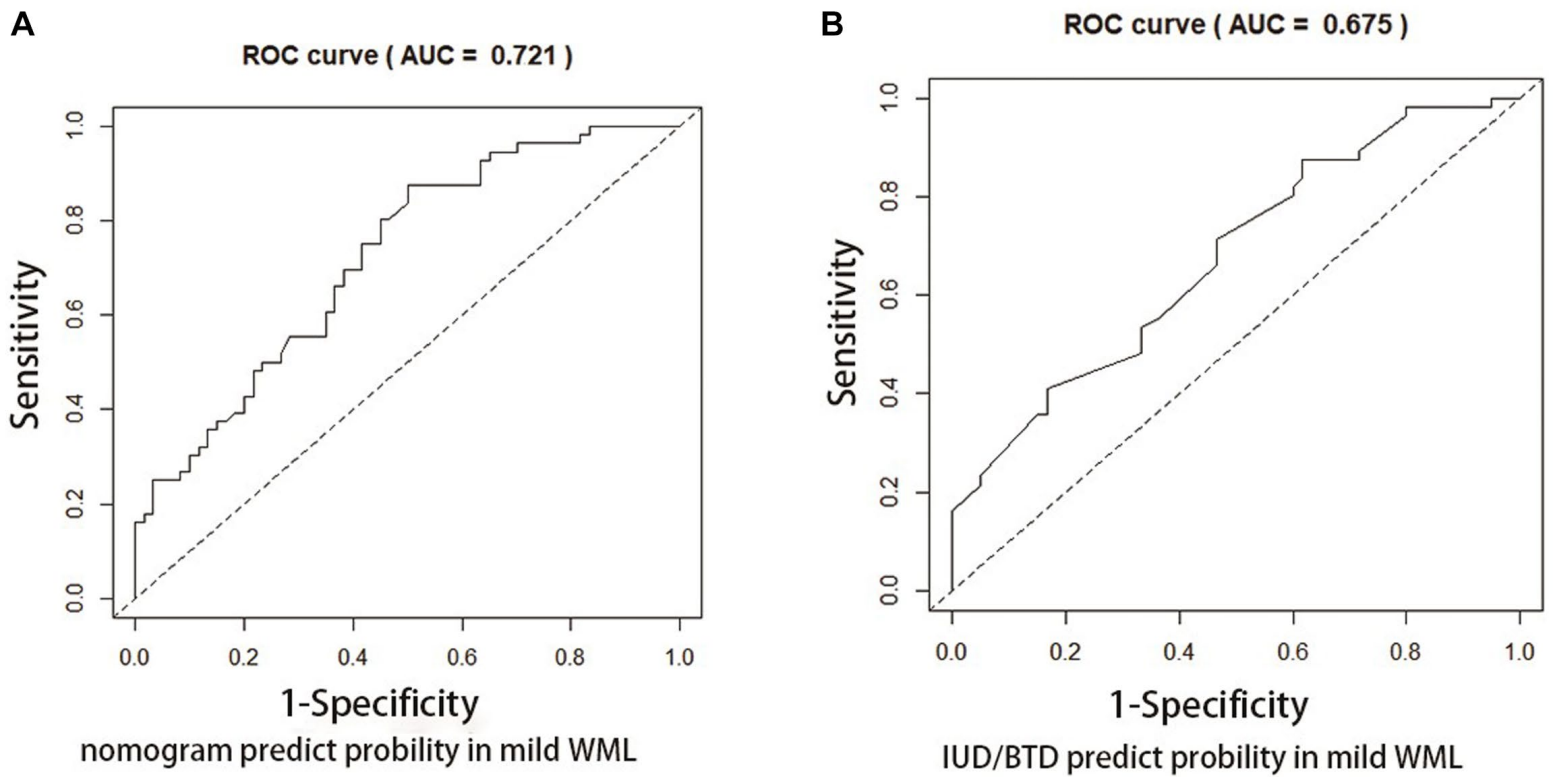
ROC curve for CSVD with MCI. ROC curves based on the nomogram **(A)** and IUD/BTD **(B)** for the probability of mild cognitive impairment in CSVD older adults.

**Figure 5:**
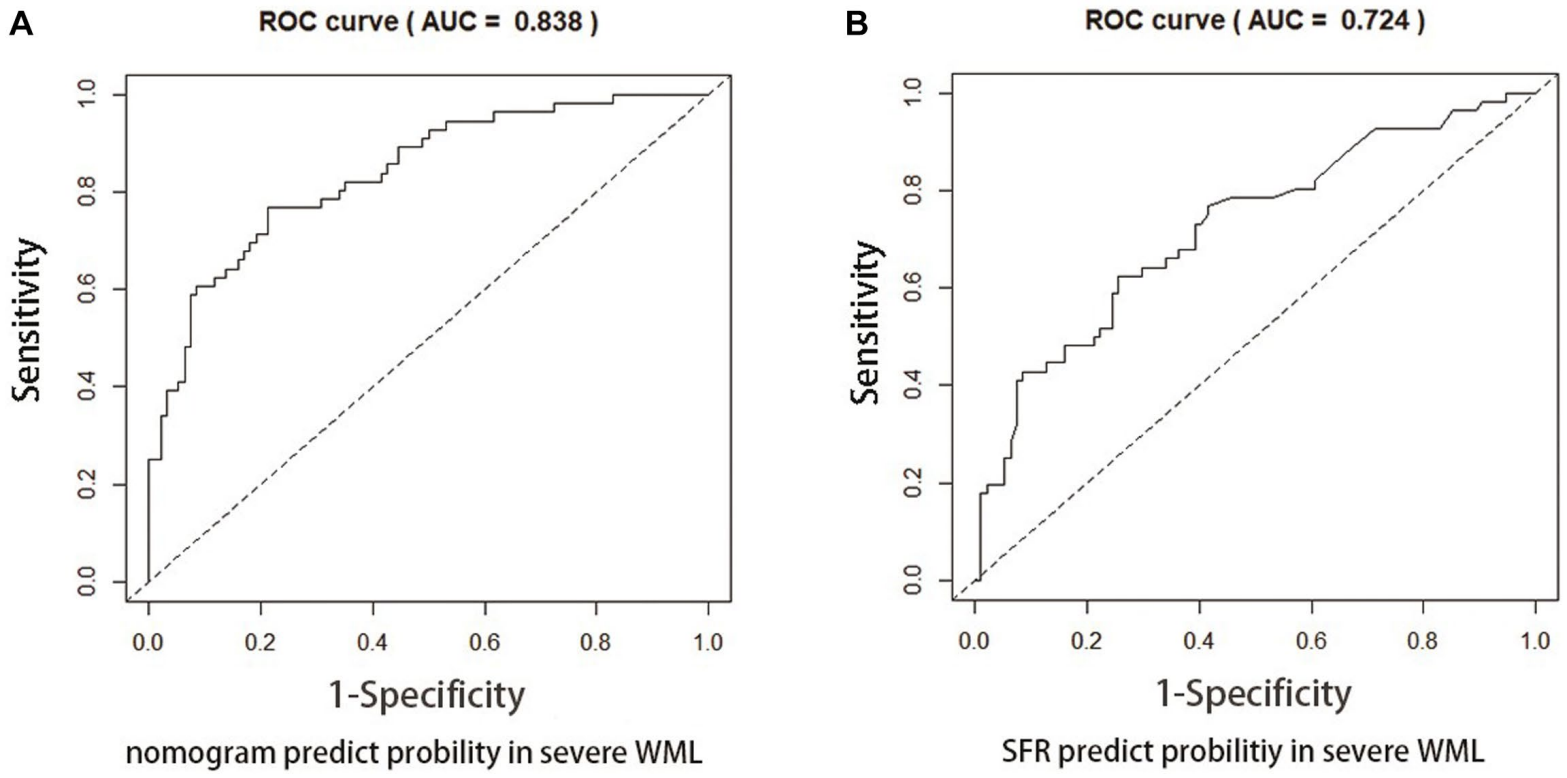
ROC curve for CSVD with dementia. ROC curves based on the nomogram **(A)** and SFR **(B)** for the probability of dementia in CSVD older adults.

Figures 4, 5 present Receiver Operating Curves (ROC) for CSVD with MCI and dementia. The results demonstrated that the imaging data all had high AUC values in both CSVD with MCI group (AUC = 0.721) (Figure 4A) and CSVD with dementia group (AUC = 0.838) (Figure 5A). The parameters with a higher global performance were two indicator of MRI linear measurements (IUD/BTD and SFR), which showed a higher predictive ability. IUD/BTD demonstrated better discriminative ability for the prediction of MCI (AUC = 0.675) (Figure 4B), and SFR demonstrated a better predictive ability for dementia than other single factors (AUC = 0.724) (Figure 5B).

## 4 Discussion

In this study, we introduced a linear brain measurement and two routine imaging examinations (carotid ultrasound and TCD) for the identification of cognitive impairment in patients with CSVD. CSVD patients with cognitive decline exhibited more severe vascular lesions or pronounced brain atrophy on imaging compared to those without cognitive impairment. In addition, with increased degree of cognitive impairment, the brain atrophy and vascular lesions showed progressive worsening. This study included two novel findings. Firstly, Crouse score and IUD/BTD were independent risk factors for MCI in CSVD patients, and independent risk factors for dementia in CSVD were Crouse Score, MCAPI, IUD/BTD, and SFR. Secondly, among these risk factors, IUD/BTD had a better predictive ability for mild cognitive impairment in CSVD older adults. SFR had a better ability to predict the onset of dementia in CSVD patients. The linear brain measurements could be an excellent cognitive screening tool for patients with CSVD.

Cognitive impairment is very common in elderly patients with CSVD (20). This situation calls for concern and needs to be addressed, regardless of the degree of severity. CSVD patients with Hypertension were more likely to have dementia, this is consistent with previous studies (21). Another result of participant characteristics was that lower LDL-C concentrations were associated with an increased risk of dementia. Lower ability of daily living could result in insignificant lack of dietary intake and nutrition in patients with dementia, which might be correlated with the reduction of LDL-C (22).

Previous studies showed that brain atrophy and change of hemodynamics were both associated cognitive decline in CSVD patients (23, 24), but their exact relationship is poorly defined. Compared to CSVD patients with MCI, patients with dementia similarly have more significant change in brain structure (linear index) and function (changes in blood flow) in our study. Among the patients with MCI, only Crouse score and IUD/BTD exhibit a significant difference compared to control. However, significantly worse neuroimaging results were present in patients with dementia, which included Crouse score, MCAPI, VBWI, MTV, and BFR, SFR, IUD/BTD. These indexes reflect cerebrovascular lesions, cortical atrophy, ventricular enlargement and hippocampal and temporal lobe atrophy. Therefore, more severe and extensive brain atrophy and vascular lesions existed in the patients with dementia implied that structure and function of brain worsened with the increasing extent of cognitive decline in CSVD patients.

In our study, the combination of Crouse score and IUD/BTD could predict mild cognitive impairment in CSVD, and the prediction accuracy reaches 72.1%. The Crouse score is a classic indicator of arteriosclerosis, which associated with the degree of carotid atherosclerosis (25). Consistent with previous studies, arteriosclerosis may be directly involved in cognitive impairment in older adults (26). As one of the linear brain measurement parameters, IUD/BTD had the greatest predictabilities for MCI in the elderly subjects with CSVD, achieving an AUC of 0.675. IUD/BTD reflects the atrophy of the temporal lobe and hippocampus (27). As a marker of neurodegeneration, the hippocampal volume is a well-known predictor of dementia in elderly subjects (28). Thus, our results indicated that hippocampal and temporal lobe atrophy were still the most important predictors of mild cognitive impairment in CSVD patients.

In the dementia group of the present study, the combination of Crouse Score, MCAPI, IUD/BTD, and SFR is able to accurately predict CSVD with dementia, and the prediction accuracy reaches 83.8%. Similar to the Crouse Score, MCAPI is an indicator of small arteriosclerosis and small vessel lesions. The increase of PI values may be representative of more extensive cerebrovascular lesions, while some scholars believe that PI values can predict worsening of cognitive function (29), this is thus a foreseeable result. The brain atrophy of the dementia patients is often more serious than that of the MCI patients (30). Undoubtedly, therefore, IUD/BTD still provide strong predictive power for dementia risk in CSVD patients. Of particular interest is SFR, as a single indicator with high predictive power, achieved an AUC of 0.724. As another linear brain measurement parameter, SFR is typically used as a measure of external atrophy (31). Kato et al. (32) indicated a larger maximum width of the Sylvian fissure, more likely indicating sub-cortical atrophy of the temporal lobe. Jeong et al. (33) found that SFR had a positive statistically significant correlation with brain atrophy in the most linear indexes. Similarly, a report showed that the enlargement of the Sylvian fissure and atrophy of the temporal lobe indicates memory deterioration (34). There were also other studies suggested that white matter hyperintensity volume and cortical thinning both worsen with age and were associated with Sylvian fissure (35), and Sylvian fissure width and several indices of brain atrophy were associated with decreased survival after age 85 (36). Furthermore, the latest study showed that SFR is an excellent indicator for the screening of cognitive decline among females (12). SFR could be an objective and accessible index of dementia in elderly CSVD patients.

In China, Imaging modalities such as Ultrasound and CT have been popularized in primary healthcare facilities (37, 38). Many older people visit these facilities for symptoms like dizziness and headache, and they and their families are more willing to undergo neuroimaging to exclude neurological diseases. However, a significant number of community/non-polyclinic doctors in China do not receive training on cognitive assessment tools (39). This lack of training, combined with the low attention and uncooperativeness of patients and their families toward the diagnosis of dementia, may contribute to the low detection rate of cognitive impairment (40). As a result, there is a higher prevalence of Mild Cognitive Impairment (MCI) in the rural population compared to the urban population in China (41). Patients residing in rural areas of China often exhibit lower levels of education. Previous studies have extensively explored the impact of education on cognitive abilities in later stages of life (42). It has been observed that education level significantly influences the results of quantitative cognitive assessment methods such as MOCA and MMSE, particularly among individuals with limited education (43). Additionally, certain studies have suggested that the language test component of MOCA may not be suitable for individuals with lower educational backgrounds (44). In such circumstances, linear brain measurements based on CT or MRI may be a more pragmatic method for screening cognitive dysfunction in a rural patient population with barriers to specialty care. In our study, linear brain measurement parameters (IUD/BTD and SFR) demonstrated good predictive power in identifying MCI and dementia in elderly subjects with Cerebral Small Vessel Disease (CSVD). Therefore, linear brain measurement appears to be a more suitable and novel method for screening cognitive impairment in aged CSVD patients in primary healthcare facilities. It is also worth considering its further promotion among the rural population.

The advantage of this study is that the subjects are all CSVD patients aged 75 years and over, which is one of the groups with the highest risk of dementia. (1) The studies of linear brain measurements related to cognitive dysfunction in elderly CSVD patients have rarely been reported before. (2) In this study, we conducted a comprehensive assessment of brain function and structure in CSVD patients, including cognitive evaluation and brain linear measurement based on MRI. In addition, TCD and carotid ultrasound were used to provide accurate assessment of cerebrovascular hemodynamics. (3) The present study identified risk factors and novel predictors associated with different degrees of cognitive impairment in CSVD.

The major limitation of this study is its cross-sectional design. While it is evident that there are associations between brain structure, cerebrovascular hemodynamics, and cognitive impairment in elderly individuals with CSVD, we cannot determine the temporal order. Additionally, the metrics used in this study were manually obtained and require further data to assess their reproducibility and accuracy. Furthermore, the sample size of this study is limited, and further investigations are required to assess the benefits of this methodology through a comparative analysis with established structural indicators of cerebral small vessel disease (CSVD), such as lesion volume, lacunes, white matter hyperintensities, cerebral microbleeds, and enlarged perivascular spaces, which would be useful covariates to include in statistical analyses. Given that linear brain measurements were a modest predictor of cognitive dysfunction, further clinical testing or referral to specialty should be followed to further confirm the diagnosis.

## Data availability statement

The raw data supporting the conclusions of this article will be made available by the authors, without undue reservation.

## Ethics statement

The studies involving humans were approved by the Ethical committee of the First Affiliated Hospital of Soochow University. The studies were conducted in accordance with the local legislation and institutional requirements. The participants provided their written informed consent to participate in this study.

## Author contributions

JW: Data curation, Formal analysis, Investigation, Methodology, Software, Visualization, Writing – original draft. JL: Data curation, Investigation, Methodology, Resources, Software, Visualization, Writing – original draft. MH: Data curation, Investigation, Methodology, Writing – original draft. ZS: Data curation, Investigation, Writing – original draft. LD: Data curation, Investigation, Writing – original draft. HT: Data curation, Investigation, Writing – original draft. YW: Conceptualization, Data curation, Formal analysis, Funding acquisition, Investigation, Methodology, Project administration, Resources, Supervision, Validation, Writing – review & editing. ZZ: Conceptualization, Data curation, Formal analysis, Funding acquisition, Investigation, Project administration, Resources, Supervision, Writing – review & editing.
